# Characteristics of children with ataxic cerebral palsy

**DOI:** 10.1186/s12887-025-05681-x

**Published:** 2025-04-29

**Authors:** Katina Pettersson, Mette Johansen, Reidun Jahnsen, Elisabet Rodby-Bousquet

**Affiliations:** 1 Centre for Clinical Research, Uppsala University - Region Västmanland, Västerås, Sweden; 2 Department of Clinical Sciences Lund, Ortopaedics, Lund University, Lund, Sweden; 3 Department of Childhood and Adolescent Medicine, Aalborg University Hospital, Aalborg, Denmark; 4 Norwegian Quality and Surveillance Registry of Cerebral Palsy (NorCP), Department of Clinical Neurosciences for Children, Oslo University Hospital, Oslo, Norway; 5 Research Center for Habilitation and Rehabilitation Models and Services (CHARM), Institute of Health and Society, University of Oslo, Oslo, Norway

**Keywords:** Cerebral palsy, Ataxia, Child, Adolescent, Brain damage, Chronic

## Abstract

**Background:**

To compare the characteristics, functional levels, and comorbidities of children with ataxic cerebral palsy (CP), with those of children with other CP subtypes.

**Methods:**

A cross-sectional study of children with CP born between 2000 and 2019 as reported in the Scandinavian national CP follow-up programmes and quality registries. Data for age, sex, levels of the Gross Motor Function Classification System (GMFCS), the Manual Ability Classification System (MACS), the Communication Function Classification System (CFCS), epilepsy, intellectual disability, and pain were extracted.

**Results:**

There were 302 children (3.9%) with ataxic CP and 7336 children (96.1%) with other subtypes. Children with ataxic CP differed significantly from other subtypes, with a greater proportion classified in GMFCS II (37.7% vs. 15%), MACS II (41.4% vs. 24.8%), and CFCS II (24.7% vs. 10.5%), more girls (50.7% vs. 41.7%), school-aged (47% vs. 41.6%), adolescents (33.4% vs. 25.4%), or had an intellectual disability (51.2% vs. 38.4%), but the prevalence of pain and epilepsy was similar between the subtypes.

**Conclusions:**

Children with ataxic CP have different characteristics and functional levels than children with other subtypes. We recommend a thorough examination of motor performance, communication, and intellectual disability to meet the individual needs of children with ataxic CP.

## Clinical trial

Not applicable.

## Background

Cerebral palsy (CP) is the most common childhood-onset lifelong condition, with a prevalence of 1.6/1000 in high-income regions in Europe, Australia, and North America, and around 3.4/1000 in low-, and middle-income countries [1]. CP is classified as spastic, dyskinetic, or ataxic CP depending on neurological signs [2]. Ataxic CP is the least common neurological subtype, with a prevalence of approximately 0.1 to 0.3/1000 [3, 4], corresponding to approximately 3.8% of all children with CP [5]. Ataxic CP is a heterogeneous group of cerebellar injuries, diseases, and genetic disorders [6, 7]. The gestational age of children with ataxic CP has shown that they are predominantly born late in the pregnancy (weeks 32 to 36) [8] or at full term (weeks 37 to 42) [5, 8]. Ataxic CP can cause several motor difficulties, and the core symptom is a lack of or loss of coordination, which causes difficulties with posture, movement, and impaired ability for motor planning [9]. Even though children with ataxic CP are reported to have better motor performance, they also have reduced dynamic trunk control affecting daily activities [9], and approximately 10% have no walking ability [10]. Ataxia can also cause additional problems in other motor systems, such as speech and vision [11]. Speech disorders occur in more than half of all children with CP, and communication difficulties seem to be more common in children with ataxic CP [6, 12].

Individuals with CP have a considerably greater risk of intellectual disability compared with the general population [13]. Intellectual disability is reported in 30–50% of the children with CP [8, 14] and is associated with more severe motor impairments [8]. Findings reported for children with ataxic CP are inconclusive, with some reports of a higher incidence of intellectual disability [5], while others report a similar prevalence compared to other subtypes [6]. In addition, the risk of epilepsy is considerably greater in children with CP than in the general population [13] and has been reported for 25–40% of the children with CP [8, 13]. However, it is unclear whether there is a difference in the prevalence of epilepsy between children with ataxic CP and those with other CP subtypes [6]. The prevalence of pain in children with CP is associated with the severity of motor impairment, but the differences between CP subtypes are unclear [15].

Most studies have focused on children with spastic CP and, more recently, on those with dyskinetic CP. The knowledge base of ataxic CP is still scarce and mostly based on data from smaller registries, local hospitals, or specific regions. Although ataxic CP is considered distinct from other forms of CP [6], there are difficulties in making a diagnosis [4], as it is highly heterogeneous [16]. The small group size of children with ataxic CP has been considered a challenge, and collaboration between population-based registries has been suggested to reduce the knowledge gap [16]. Therefore, we combined data from three national CP registries with high enrolment rates, to provide a more comprehensive picture of children with ataxic CP.

The aim of this study was to compare the characteristics (*age*, *sex*), functional levels (*gross motor function*, *manual ability*, *communication*), and comorbidities (*intellectual disability*, *epilepsy*, *pain*) of children with ataxic CP, compared to children with other CP subtypes.

## Methods

### Study design and settings

This was a cross-sectional observational study based on data from three Scandinavian CP follow-up programmes and quality registries: the Swedish CP Follow-up Program and Quality Registry (CPUP) [17], the Norwegian Quality and Surveillance Registry of CP (NorCP) [18], and the Danish CP Follow-up Program (CPOP) [19]. These registries and follow-up programmes have been active in Sweden since 1994 (with National data from 2007), in Norway since 2006, and in Denmark since 2010, and are fully integrated into the respective country’s specialized paediatric health care services. Therefore, enrolment levels are high and include > 95% of all children with CP in Sweden [20], 93% in Norway [18], and 82% in Denmark [19]. All countries use measures according to standardized protocols, which are similar across countries [21]. All the data are reported in the registries by paediatric physiotherapists, occupational therapists, psychologists, and neurologists. In all three countries, children are followed systematically throughout childhood with repeated clinical examinations depending on age and GMFCS level.

### Participants

We included data on all children with CP reported in the CP registries from Sweden (2007), Norway (2006) and Denmark (2010) until 2019, and used the child’s latest registration in all our analyses. In Sweden, 4499 children were born from 2000 to 2019 (mean age 9 years, 8 months); in Norway, 1663 children were born from 2002 to 2019 (mean age 9 years, 5 months); and in Denmark, 1476 children were born from 2000 to 2019 (mean age 7 years, 1 month).

### Classifications and measurements

The inclusion and exclusion criteria for CP and subtypes were defined according to the Surveillance of Cerebral Palsy in Europe [2], and subtypes were dichotomized into ataxic CP and other subtypes (including spastic, dyskinetic and mixed type/unclassified CP). Male or female sex was based on their legally recognized sex, and age was calculated based on birth date and examination date. Age was grouped into three categories: (1) pre-schoolers (0 − 6 years); (2) school-aged (7 − 12 years); and (3) adolescents (13 − 19 years). In Norway and Denmark, children start school at 6 years old, and in Sweden, at 7 years old. We classified all children < 7 years old as pre-schoolers.

The functional levels I to V were classified according to the expanded and revised versions of the Gross Motor Function Classification System (GMFCS) [22], the Manual Ability Classification System (MACS) [23], and the Communication Function Classification System (CFCS) [24]. Intellectual disability was formally tested and classified by a psychologist or neuropaediatrician according to ICD-10 codes as Yes (IQ below 70) or No (IQ above 70). Epilepsy was reported by neuropaediatricians and coded as Yes or No. Current pain was either self-reported or proxy reported as Yes or No [25].

### Statistical analysis

Normally distributed data are presented as the means with standard deviations (SD). Differences between groups were evaluated with a Pearson chi-square test. Binary logistic regression models were used to estimate intellectual disability among the different subtypes adjusted for GMFCS, MACS, CFCS, age, and sex. The results are presented as adjusted odds ratios (OR) with 95% confidence intervals (CI). Subtypes were treated as nominal categories with dyskinesia as a reference category, whereas GMFCS, MACS, and CFCS were treated as ordinal categories using level 1 as a reference. For sex, male sex was used as the reference category. Age was used as a continuous variable in the regression analyses. R^2^ was used as a goodness-of-fit measure to indicate the percentage of the variance in the dependent variable explained by the independent variables collectively. The significance level was set to *p* < 0.05. IBM SPSS Statistics for Windows version 28 (IBM Corp., Armonk, NY, USA) was used for all analyses.

## Results

In the present study, there were 302 children (3.9%) with ataxic CP corresponding to a prevalence of 0.082/1000, and 7336 children (96.1%) with other subtypes. Spastic CP was the most common subtype, with 6264 children (82.0%), followed by 774 children (10.1%) with dyskinetic CP and 298 children (3.9%) with mixed types/unclassified CP, Table 1. Children with ataxic CP were slightly older at the most recent examination (mean age 10 years, 5 months [SD 4 years, 1 month]) than were children with other subtypes (mean age 9 years, 1 month [SD 4 years, 5 months]).

Compared with children with other subtypes, children with ataxic CP had significant differences in all characteristics, functional levels, and intellectual disability, but similar prevalence of epilepsy and pain. In children with ataxic CP, there was a significantly greater proportion classified as level II for GMFCS (37.7% vs. 15%) and MACS (41.4% vs. 24.8%) and less in GMFCS and MACS level V, than in children with other subtypes. There was a greater variability in communication for children with ataxic CP, with a higher proportion of children in CFCS levels II, III, and IV, Table 1.

**Table 1:** Characteristics, functional levels, comorbidities, and missing data for children with ataxic CP and other subtypes

		Ataxia		Other subtypes		
		*n*	%	*n*	%	*p*
Total (*n* = 7638)		302	3.95	7336	96.05	
Age at examination	Pre-schooler 0–6 y	59	19.5	2418	33.0	< 0.001
	School-aged 7–12 y	142	47.0	3051	41.6	
	Adolescent 13–19 y	101	33.4	1867	25.4	
Sex	Male	149	49.3	4280	58.3	0.002
	Female	153	50.7	3056	41.7	
GMFCS	I	120	39.7	3537	48.2	< 0.001
	II	114	37.7	1098	15.0	
	III	34	11.3	540	7.4	
	IV	28	9.3	924	12.6	
	V	6	2.0	1237	16.9	
MACS	I	72	25.9	2489	35.8	< 0.001
	II	115	41.4	1728	24.8	
	III	54	19.4	925	13.3	
	IV	26	9.4	710	10.2	
	V	11	4.0	1109	15.9	
	Total	278	100.0	6961	100.0	
	Missing	24		375		
CFCS	I	70	30.8	2612	50.8	< 0.001
	II	56	24.7	540	10.5	
	III	33	14.5	528	10.3	
	IV	48	21.1	687	13.4	
	V	20	8.8	777	15.1	
	Total	227	100.0	5144	100.0	
	Missing	75		2192		
Comorbidities						
Epilepsy	Yes	65	38.0	1266	36.4	0.675
	No	106	62.0	2209	63.6	
	Missing	131		3599		
Intellectual disability	Yes	62	51.2	1008	38.4	0.005
	No	59	48.8	1617	61.6	
	Missing	181		4711		
Pain	Yes	110	37.2	2814	40.1	0.307
	No	186	62.8	4198	59.9	
	Missing	6		324		

GMFCS, Gross Motor Function Classification System; MACS, Manual Ability Classification System; CFCS, Communication Function Classification System

### Sex differences

The female − male ratio was more even in children with ataxic CP (1:0.97), compared to the other subtypes (1:1.4). The ataxic group had significantly more girls (50.7%) than did the other subtypes (41.7%), Table 1. GMFCS distribution differed between boys and girls with ataxic CP, with more boys in GMFCS II (45.6%) and more girls in GMFCS I (47.7%). There were no sex differences regarding MACS, CFCS, or any of the comorbidities between boys and girls with ataxic CP, in contrast to the other subtypes with significant differences regarding pain, Table 2.

**Table 2:** Sex differences in functional levels and comorbidities in children with ataxic CP compared with those in other subtypes

		Ataxia			Other subtypes		
		Sex			Sex		
		Male	Female		Male	Female	
		*n* (%)	*n* (%)		*n* (%)	*n* (%)	
Total		149 (49.3)	153 (50.7)	302 (100.0)	4280 (58.3)	3056 (41.7)	7336 (100.0)
				*p*			*p*
GMFCS	I	47 (31.5)	73 (47.7)	0.031	2091 (48.9)	1445 (47.3)	0.206
	II	68 (45.6)	46 (30.1)		607 (14.2)	491 (16.1)	
	III	18 (12.1)	16 (10.5)		324 (7.6)	216 (7.1)	
	IV	14 (9.4)	14 (9.2)		541 (12.6)	383 (12.5)	
	V	2 (1.3)	4 (2.6)		716 (16.7)	521 (17.0)	
	Total	149 (100.0)	153 (100.0)		4280 (100.0)	3056 (100.0)	
MACS	I	29 (21.2)	43 (25.9)	0.481	1430 (35.2)	1059 (36.6)	0.583
	II	61 (44.5)	54 (41.4)		1010 (24.8)	718 (24.8)	
	III	29 (21.2)	25 (19.4)		557 (13.7)	368 (12.7)	
	IV	13 (9.5)	13 (9.4)		425 (10.5)	285 (9.8)	
	V	5 (3.6)	6 (4.0)		644 (15.8)	465 (16.1)	
	Total	137 (100.0)	141 (100.0)		4066 (100.0)	2895 (100.0)	
CFCS	I	29 (26.1)	41 (35.3)	0.203	1540 (50.9)	1072 (50.6)	0.168
	II	28 (25.2)	28 (24.1)		302 (10.0)	238 (11.2)	
	III	22 (19.8)	11 (9.5)		304 (10.0)	224 (10.6)	
	IV	22 (19.8)	26 (22.4)		430 (14.2)	257 (12.1)	
	V	10 (9.0)	10 (8.6)		451 (14.9)	326 (15.4)	
	Total	111 (100.0)	116 (100.0)		3027 (100.0)	2117 (100.0)	
Comorbidities							
Epilepsy	Yes	31 (37.3)	34 (38.6)	0.862	726 (35.3)	540 (38.1)	0.083
	No	52 (62.7)	54 (61.4)		1333 (64.7)	876 (61.9)	
	Total	83 (100.0)	88 (100.0)		2059 (100.0)	1416 (100.0)	
Intellectual disability	Yes	30 (49.2)	32 (53.3)	0.648	583 (37.8)	425 (39.3)	0.420
	No	31 (62.2)	28 (46.7)		961 (62.2)	656 (60.7)	
	Total	61 (100.0)	60 (100.0)		1544 (100.0)	1081 (100.0)	
Pain	Yes	55 (37.9)	55 (36.4)	0.789	1571 (38.5)	1243 (42.4)	< 0.001
	No	90 (62.1)	96 (63.6)		2511 (61.5)	1687 (57.6)	
	Total	145 (100.0)	151 (100.0)		4082 (100.0)	2930 (100.0)	

GMFCS, Gross Motor Function Classification System; MACS, Manual Ability Classification System; CFCS, Communication Function Classification System

### Age differences

The majority of children with ataxic CP were school-aged (47.0%). There were significantly fewer children with ataxic CP in the youngest age group (19.5%) than in the other subtypes (33%). Additionally, there were fewer children in GMFCS II among pre-schoolers (27.1%) and more among school-aged children (42.3%). The proportion of children with MACS levels I and III was greater in older children, whereas the proportion of children with MACS levels II, IV, and V was smaller. Most children in CFCS I were school-aged or adolescents. Epilepsy was more common in the older age groups. Almost half (48.4%) of the children with ataxic CP had an intellectual disability at school age, and more than half had an intellectual disability in adolescence (58.7%). Pain prevalence was greater in the older age groups in all subtypes, Table 3.

**Table 3:** Functional levels and comorbidities in different age groups for children with ataxic CP compared with other subtypes

		Ataxia					Other subtypes			
		Age					Age			
		Pre-schooler	School-aged	Adolescent			Pre-schooler	School-aged	Adolescent	
		*n* (%)	*n* (%)	*n* (%)			*n* (%)	*n* (%)	*n* (%)	
Total		59 (19.5)	142 (47.0)	101 (33.4)	302 (100.0)		2418 (33.0)	3051 (41.6)	1867 (25.4)	7336 (100.0)
					*p*					*p*
GMFCS	I	28 (47.5)	56 (39.4)	36 (35.6)	0.203		1178 (48.7)	1538 (50.4)	821 (44.0)	< 0.001
	II	16 (27.1)	60 (42.3)	38 (37.6)			349 (14.4)	482 (15.8)	267 (14.3)	
	III	7 (11.9)	11 (7.7)	16 (15.8)			204 (8.4)	191 (6.3)	145 (7.8)	
	IV	5 (8.5)	13 (9.2)	10 (9.9)			281 (11.6)	357 (11.7)	286 (15.3)	
	V	3 (5.1)	2 (1.4)	1 (1.0)			406 (16.8)	483 (15.8)	348 (18.6)	
	Total	59 (100.0)	142 (100.0)	101 (100.0)			2418 (100.0)	3051 (100.0)	1867 (100.0)	
MACS	I	12 (22.6)	34 (25.8)	26 (28.0)	0.552		728 (32.6)	1139 (38.8)	622 (34.6)	< 0.001
	II	26 (49.1)	57 (43.2)	32 (34.4)			627 (28.1)	723 (24.7)	378 (21.0)	
	III	7 (13.2)	23 (17.4)	24 (25.8)			284 (12.7)	366 (12.5)	275 (15.3)	
	IV	5 (9.4)	14 (10.6)	7 (7.5)			235 (10.5)	261 (8.9)	214 (11.9)	
	V	3 (5.7)	4 (3.0)	4 (4.3)			358 (16.0)	444 (15.1)	307 (17.1)	
	Total	53 (100.0)	132 (100.0)	93 (100.0)			2232 (100.0)	2933 (100.0)	1796 (100.0)	
CFCS	I	6 (16.7)	33 (30.0)	31 (38.3)	0.221		661 (41.5)	1210 (56.1)	741 (53.2)	< 0.001
	II	9 (25.0)	29 (26.4)	18 (22.2)			182 (11.4)	219 (10.1)	139 (10.0)	
	III	8 (22.2)	16 (14.5)	9 (11.1)			225 (14.1)	177 (8.2)	126 (9.1)	
	IV	11 (30.6)	24 (21.4)	13 (16.0)			254 (15.9)	246 (11.4)	187 (13.4)	
	V	2 (5.6)	8 (7.3)	10 (12.3)			272 (17.1)	306 (14.2)	199 (14.3)	
	Total	36 (100.0)	110 (100.0)	81 (100.0)			1594 (100.0)	2158 (100.0)	1392 (100.0)	
Comorbidities										
Epilepsy	Yes	6 (33.3)	32 (38.1)	27 (39.1)	0.903		198 (30.8)	553 (34.6)	515 (41.7)	< 0.001
	No	12 (66.7)	52 (61.9)	42 (60.9)		445 (69.2)		1044 (65.4)	720 (58.3)	
	Total	18 (100.0)	84 (100.0)	69 (100.0)		643 (100.0)		1597 (100.0)	1235 (100.0)	
Intellectual disability	Yes	5 (38.5)	30 (48.4)	27 (58.7)	0.354	180 (34.8)		457 (36.3)	371 (43.8)	< 0.001
	No	8 (61.5)	32 (51.6)	19 (41.3)		337 (65.2)		803 (63.7)	477 (56.3)	
	Total	13 (100.0)	62 (100.0)	46 (100.0)		517 (100.0)		1260 (100.0)	848 (100.0)	
Pain	Yes	15 (26.3)	48 (34.0)	47 (48.0)	0.015	670 (29.1)		1256 (43.0)	888 (49.7)	< 0.001
	No	42 (73.7)	93 (66.0)	51 (52.0)		1634 (70.9)		1666 (57.0)	898 (50.3)	
	Total	57 (100.0)	141 (100.0)	98 (100.0)		2304 (100.0)		2922 (100.0)	1786 (100.0)	

GMFCS, Gross Motor Function Classification System; MACS, Manual Ability Classification System; CFCS, Communication Function Classification System

### Comorbidities

Both epilepsy and pain had similar frequencies to that in the other CP subtypes, Table 1. Most children with epilepsy (73.8%), also had an intellectual disability (data not shown). In total, 51.2% of the children with ataxic CP had an intellectual disability, Table 1.

Ataxic CP was associated with a greater likelihood of intellectual disability (OR 3.23, 95% CI 1.58 − 6.62) than children with dyskinetic CP when adjusted for GMFCS, MACS, CFCS, age, and sex (R^2^ = 64.8%), Table 4.

**Table 4:** Binary logistic regression analyses for having intellectual disability and CP subtypes, presented as odds ratios (OR) with 95% confidence intervals (CI)

		Intellectual disability			
		OR	95% CI		*p*
			Lower	Upper	
Subtype	DY	*ref.*			< 0.001
	SP	2.24	1.40	3.58	< 0.001
	AT	3.23	1.58	6.62	0.001
	UC	15.37	4.40	53.71	< 0.001

All values are adjusted for GMFCS, MACS, CFCS, age, and sex. GMFCS, Gross Motor Function Classification System; MACS, Manual Ability Classification System; CFCS, Communication Function Classification System. CP subtypes: DY, dyskinetic; SP, spastic; AT, ataxic; UC, mixed type/unclassified CP

## Discussion

We found significant differences between children with ataxic CP and children with other CP subtypes for all characteristics, functional levels, and intellectual disability but not for epilepsy or pain. Motor function differed for children with ataxic CP, with more children in GMFCS and MACS II than in level I, which is usually seen in other subtypes. Interestingly, we found that girls with ataxic CP had better gross motor function than boys. We also observed a greater variability in communication, as children with ataxic CP were evenly spread across all CFCS levels.

The prevalence of ataxic CP in our study was 3.9%, consistent with previous findings [5, 26]. Within the ataxic spectrum, the prevalence of ataxic CP has apparently been stable since at least the 1980s [3] and is not decreasing, in contrast to the general trend for CP, potentially because ataxic CP may originate from genetic causes [7], or by the fact that most children with ataxic CP are born at term [5, 8]. We found that the distribution of GMFCS levels differed significantly between children with ataxic CP and those with other CP subtypes. This has not been identified in previous studies, where investigators grouped the GMFCS levels into two or three groups and did not analyse them separately [5, 8]. However, a high proportion of children with ataxic CP classified in GMFCS level II has previously been reported by Westbom et al. [4], and Påhlman et al. [8]. According to Beckung [10], intellectual disability is an important explanatory factor for walking ability in all CP subtypes. This might reflect the high prevalence of intellectual disability we found in children classified in GMFCS level II. It may also be due to reduced balance in children with ataxic CP [9], where a difference between GMFCS I and II is the use of a railing to walk up and down stairs and balancing. In our present study, slightly more than every tenth child with ataxic CP, mostly pre-schoolers, did not walk. It is possible they may still develop the skill, given some of them were still very young. Considering that many of the children with ataxic CP also have an intellectual disability, learning and development may take longer [8]. Some studies have shown greater proportions of non-walkers, ranging from 13 to 27% [5, 6], whereas others have shown few or no non-walkers (6% to 0) [4, 8]. This range of non-walkers between studies is difficult to explain. It may be due to mixed populations analysing children with ataxic and hypotonic CP together, different sample sizes, or differences in age when assessing walking ability. Based on these data, one cannot assume that all children with ataxic CP will walk, but regardless of their mobility, they, like all children with CP, require regular assessments of motor function, training, and follow-up, especially during younger years.

Most children with ataxic CP had better hand function (MACS levels I to III), compared to the other CP subtypes, where more children were classified with MACS V (16% vs. 4%). A significantly higher proportion of the children with ataxic CP were classified with MACS II with limitations to speed or quality compared to children with other subtypes. Also, Påhlman et al. reported that more than half were classified in MACS level II [8]. When comparing hand function to the large population reported by Horber et al. [5], we had fewer individuals in the MACS I and II levels (62% versus 75%) and, conversely, more individuals in level III (18% versus 10%). Påhlman et al.’s data stand out, as almost one-third of their population was classified in MACS levels IV or V [8], while Horber et al. [5] reported a distribution similar to that found in our study. As intellectual disability is described as the single most explanatory factor for walking ability [10], the probability that this might also be the case for hand function is high. As for gross motor ability, fine motor function is dependent on a stable trunk, so reduced balance can also affect fine motor performance [9]. Despite this subtype´s abnormal patterns of movements (due to the brain damage itself), and problems with hand function, (such as past pointing, tremor, and low muscle tone) [5], it seems to affect hand function to a lesser degree, than for the other CP subtypes.

As Levy et al. (6) observed, children with ataxic CP had significantly more communication impairments than did those with other CP subtypes. We also found greater variability in communication ability within the group of children with ataxic CP than in other studies [5, 8, 10] but also in comparison to children with other CP subtypes [14]. A likely reason for this difference is the role played by the cerebellum in language production and processing [27]. However, while we have used the CFCS, others have used alternative speech scales (The Viking Speech Scale) [5, 8], thus making true comparisons difficult. As for other motor skills, speech is intricately intertwined with intellectual disability, as research has found that children with less cognitive ability also have less communication ability [14]. Nevertheless, communication is essential for participation and independence in all aspects of life, from childhood to adulthood [28].

Consistent with previous studies [4, 8], we found a more even female–male ratio in children with ataxic CP, with more girls than in those with other CP subtypes. Notably, there were significant differences in GMFCS levels between the sexes, with better gross motor function in girls than in boys. To our knowledge, this has not been reported before. A potential explanation could be better balance in girls. While reduced balance is one of the cardinal symptoms in ataxic CP, also pre-school girls without CP have better balance compared to boys [29, 30]. When considering the entire CP population, others have not found any significant sex differences [31], except for pain, which is significantly more common in girls [15]. However, we did not find this difference in ataxic CP.

There was a greater proportion of school-aged and adolescent children with ataxic CP than in other subtypes. This greater proportion might indicate difficulties in differentiating ataxic CP from other subtypes [5, 32] and that children with ataxic CP need to be older to receive an accurate diagnosis. In future studies, it could be of interest, to look at what age children with ataxic CP receive their diagnosis. The distribution of MACS levels at different ages showed a trend towards better hand function (MACS I) with older age. Moreover, the proportion of those at CFCS I increased in older age groups, indicating their better communication. A possible explanation could be the interplay between cognition and motor development, and since many of the children who have ataxia also have an intellectual disability, learning and development may take longer [8]. Or is it simply due to age? That children with atactic CP need longer time and practice, to achieve motor milestones. Studies show that individuals with ataxia benefit from intense rehabilitation treatments, that includes interventions aimed at both activating balance control and multi-joint coordination [33].

The cerebellum is not only involved in ataxia, but may also influence cognitive functions [27]. Consistent with Påhlman et al. [8], we found that intellectual disability was significantly more common in children with ataxic CP compared to all other subtypes, and increased in older age groups. Others have explained the increase with augmented testing over time, as abilities develop later and cannot be assessed until the child reaches school age [8]. This increase might be explained by the later maturity of both the prefrontal cortex and the cerebellum, as many cognitive tasks that require the prefrontal cortex also require the cerebellum [34]. Stadskleiv et al. reported a negative correlation between age and intellectual disability [14], as older children showed progress in their abilities. Nevertheless, the increase in raw scores with age did not match the expected increase. It is imperative to base the diagnosis on a comprehensive individual assessment, not only on IQ, but also on the individual’s social, executive, and adaptive functioning [14].

Like Levy et al. [6], we found no differences between groups on epilepsy, in contrast to others [5, 8, 35]. This discrepancy could be due to sample size or statistical methods. Epilepsy seemed to increase with age. Comorbidities, such as epilepsy, can sometimes be more disabling than the motor disorder itself [35].

### Strengths and limitations

There are several limitations to this study. This was a cross-sectional study that reflects the status at a particular point in time and not longitudinal changes. Some variables had a high proportion of missing data, such as for intellectual disability. This is a major concern and there is ongoing work in all three countries to improve the assessment of intellectual disability and cognitive functioning for individuals with CP [36]. Also, we did not know if the children with epilepsy had ongoing treatment, even though they all had their diagnosis set by a neuropaediatrician. Ataxic CP is rare and considered difficult to differentiate, thus making it likely that this rare subtype remains under-recognized [4]. A strength of this study is the large study population enrolled in well-established national population-based programmes. A high level of enrolment [17–19] reduces the risk of selection bias and allows for the differentiation and comparisons of functional levels, sex, and age groups.

## Conclusion

Children with ataxic CP have significantly different characteristics, functional levels, and a higher prevalence of intellectual disability than children with other subtypes. Therefore, we recommend a thorough examination of motor performance, communication, and intellectual disability to meet the individual needs of children with ataxic CP. Regional and global collaboration to detect differences or similarities in this small subtype within the CP spectrum is warranted. In addition, knowledge of aetiology would provide more insight into this group of children. We also recommend examining this group from a sex and age perspective, as we found that these aspects differ for this subtype compared with the other subtypes.

Ataxia not only influences all areas of daily life but is also associated with significant economic costs and decreased quality of life [3]. As the cerebellum, with its cardinal role, controls and influences affective regulation, cognitive processing, and linguistic functions [27], this makes us aware of the need to thoroughly examine all these aspects to obtain in-depth knowledge of each child’s needs. This Scandinavian collaboration contributes to increased knowledge about children with ataxic CP and helps us understand the smallest, and sometimes debated CP subtype, as there is an ongoing discussion in the medical-/ research community about the definition of ataxic CP [16]. This knowledge can hopefully contribute to guiding everyday clinical practice and the individual’s everyday life by tailoring interventions based on the needs of children with ataxic CP.

## Data Availability

The data that support the findings of this study are available from the Scandinavian CP registries. The following restrictions apply; requests to access the datasets are subject to ethical approval in each country according to national regulations and legislations.
